# Reassortment events in the evolution of hantaviruses

**DOI:** 10.1007/s11262-018-1590-z

**Published:** 2018-07-25

**Authors:** Boris Klempa

**Affiliations:** 1 Biomedical Research Center, Institute of Virology, Slovak Academy of Sciences, Bratislava, Slovakia; 2 Institute of Virology, Charité University Hospital, Helmut-Ruska-Haus, Berlin, Germany

**Keywords:** Hantavirus, Reassortment, Evolution

## Abstract

Hantaviruses (order *Bunyavirales*, family *Hantaviridae*), known as important zoonotic human pathogens, possess the capacity to exchange genome segments via genetic reassortment due to their tri-segmented genome. Although not as frequent as in the arthropod-borne bunyaviruses, reports indicating reassortment events in the evolution of hantaviruses have been recently accumulating. The intra- and inter-lineage reassortment between closely related variants has been repeatedly reported for several hantaviruses including the rodent-borne human pathogens such as Sin Nombre virus, Puumala virus, Dobrava-Belgrade virus, or Hantaan virus as well as for the more recently recognized shrew-borne hantaviruses, Imjin and Seewis. Reassortment between more distantly related viruses was rarely found but seems to play a beneficial role in the process of crossing the host species barriers. Besides the findings based on phylogenetic studies of naturally occurring strains, hantavirus reassortants were generated also in in vitro studies. Interestingly, only reassortants with exchanged M segments could be generated suggesting that a high degree of genetic compatibility is required for the S and L segments while the exchange of M segment is better tolerated or is particularly beneficial. Altogether, the numerous reports on hantavirus reassortment, summarized in this review, clearly demonstrate that reassortment events play a significant role in hantavirus evolution and contributed to the currently recognized hantavirus diversity.

## Introduction

First reports that genome segment exchange, in other words genetic reassortment events, could have been occurring in the evolution of hantaviruses are rather old and emerged soon after the discovery of hantavirus cardiopulmonary syndrome-causing Sin Nombre virus (SNV) in the United States [1, 2]. Until recently, reassortment (more precisely its absence) was even used as one of the taxonomical species demarcation rules. In general, it was considered as rather exceptional process in the hantavirus evolution. However, reports on conflicting findings in the segment-specific evolutionary trees suggesting reassortment have been recently accumulating across both the “old” rodent-borne hantaviruses as well as the more recently recognized new hantaviruses found in non-rodent hosts. In light of these findings, it seems that reassortment is more common among hantaviruses than previously reported. Moreover, it is highly likely that the recent progress in the utilization of next-generation-sequencing (NGS) technologies leading to massive increase of full genome sequences will bring more findings in this area, too. It is, therefore, time to review and summarize the current knowledge on reassortment findings in the recently established family *Hantaviridae*.

Hantaviruses (order *Bunyavirales*, family *Hantaviridae*) are enveloped, single stranded RNA viruses with segmented genome of negative polarity. The genome is composed of three segments, small (S) segment encoding nucleocapsid protein (N protein) [3], medium (M) segment encoding glycoprotein precursor (GPC) co-translationally cleaved into the envelope glycoproteins Gn and Gc [4], and large (L) segment encoding the L protein primarily serving as the viral RNA-dependent RNA polymerase (RdRp) [5].

Virus entry into cells is mediated by binding to a cell surface receptor. Integrins are considered to be the main receptors for hantaviruses at least in vitro [6–8] but other molecules, complement decay accelerating factor (DAF) [9], and globular heads of complement C1q receptor (gC1qR) [10], were reported to mediate hantavirus infection in cultured cells, too. The entry then proceeds through clathrin-dependent endocytosis shown for the prototypical Hantaan virus (HTNV) [11] and/or other pathways including micropinocytosis, clathrin-independent receptor-mediated endocytosis, or other routes [12, 13]. Viral particles are then trafficked to late endosomes. Low Ph-triggered, Gc-mediated virus cell membrane fusion releases viral genetic material into the cytoplasm. Transcription of the viral mRNAs includes the process of cap-snatching and involves localization of N and L proteins to cytoplasmic processing bodies (P bodies) where they use the caps of host mRNAs destined for degradation [14]. The hantavirus RNA synthesis is initiated by the prime-and-realign mechanism [15]. The endoplasmic reticulum-Golgi intermediate compartment (ERGIC) is considered to be the site of viral replication [16]. The virions are assumed to bud into the cis-Golgi and then transported to the plasma membrane for release, presumably via recycling endosomes [17].

When transmitted to humans, hantaviruses can cause severe disease. Transmission occurs usually through inhalation of aerosolized rodent excreta and rarely via biting by infected animals [18], but the human intestinal tract is a possible entrance port, too [19]. Hantaviruses present on the American continent, such as SNV or Andes virus (ANDV) cause hantavirus (cardio)pulmonary syndrome. HTNV and Seoul virus (SEOV) in Asia and Puumala virus (PUUV) and Dobrava-Belgrade virus (DOBV) in Europe are most common hantaviruses causing hemorrhagic fever with renal syndrome. Both diseases share the main pathogenetic mechanisms involving changes in blood coagulation, vasodilatation, and disturbances in the barrier function of the capillaries, resulting in capillary leakage and inflammatory processes in the affected organs [18].

Hantaviruses produce chronic and asymptomatic infection in their reservoir hosts, small mammals. Besides with hantaviruses typically associated rodents, other small mammals such as shrews, moles, and bats were identified as hantavirus reservoir hosts during the last decade [20–22]. Very recently, using a large-scale meta-transcriptomic approach, hantavirus-related sequences were identified even in reptiles, ray-finned fish, and jawless fish [23]. Although there are accumulating exceptions, hantaviruses are in general considered to be host-specific. A particular hantavirus is usually transmitted only by one or few closely related host species. This association is at least partially reflected also in their phylogeny, particularly among the rodent-borne hantaviruses. Therefore, hantaviruses have been considered to have co-evolved with their hosts over millions of years [24]. Recent phylogenetic analyses including the more recently discovered shrew-, mole-, and bat-borne hantaviruses revealed a complex evolutionary history where not only virus-host co-divergence but also cross-species transmission and ancient reassortment events played a role. Furthermore, these analyses also suggest that shrews, moles, or bats might have been the hosts of ancestral hantaviruses [25–28].

Reassortment is defined as exchange of gene segments between viruses that co-infect the same cell, which can result in the formation of progeny viruses that are genetically distinct from both parental viruses. Therefore, reassortment can create viral progeny conferring important fitness advantages. On the other hand, successful reassortment between two parental strains during co-infection requires a high degree of genetic compatibility including intricate packaging signals and RNA–RNA and/or RNA–protein interactions [29].

Reassortment is particularly well known for influenza A virus where it is associated with the antigenic shift and emergence of new pandemic strains [30]. However, all viruses with segmented genomes possess the capacity to exchange genome segments. Reassortment has been well documented for several other pathogenic viruses such as reoviruses, arenaviruses, or bunyaviruses [29, 31, 32]. Clearly, the ability of important human pathogens to reassort not only has implications for their ongoing evolution but can also lead to changes in their virulence and transmission efficiency and, therefore, has impact on public health.

### Reassortment within the order *Bunyavirales*

Bunyaviruses (order *Bunyavirales*) with their tri-segmented genome are obvious candidates for reassortment playing role in their evolution. Indeed, reassortment seems to be rather frequently reported within the order, especially within the family *Peribunyaviridae* (former genus *Orthobunyavirus*). Particularly interesting is the fact that there are frequent reassortment events found between distinct viruses (i.e., heterotypic reassortment). Briese et al. [32] even suggested that most if not all currently recognized bunyaviruses in fact represent reassortants of existing or extinct viruses. The high frequency of reassortment might be explained by the fact that many of these viruses are arthropod-borne viruses (arboviruses) and are, therefore, capable of alternately replicating in hematophagous arthropods and vertebrates. Dual infections of arthropod hosts provide considerable opportunity for reassortment of the genome segments. Particularly mosquitoes and culicoids (unlike ticks) feed frequently, providing a greater opportunity for dual infections in them as well as in their vertebrate hosts. Another interesting phenomenon is the super-infection resistance which may prevent secondary infection by closely related bunyaviruses and thereby reduce the frequency of co-infections. However, it may actually promote opportunities for segment reassortment between more distantly related bunyaviruses [32].

Numerous examples of natural occurrences of reassortment can be found across the order and were recently systematically reviewed by Briese et al. [32]. For instance, Jatobal virus and Iquitos virus of the Simbu serogroup of orthobunyaviruses are both reassortants containing S and L segments of Oropouche virus and a unique M segment of a yet unrecognized Simbu serogroup virus [33, 34]. Complex reassortment scenarios were reported also for Shamonda and Schmallenberg viruses [35]. Similarly, several viruses of the group C orthobunyaviruses such as Apeu, Murutucu, and Itaqui, represent reassortants with various combinations of segments from Marituba, Caraparu, and Oriboca viruses [36]. Another well-documented example within the Bunyamwera serogroup viruses is the hemorrhagic fever causing Ngari virus (and its isolate Garissa virus) which is a reassortant containing S and L segments of Bunyamwera virus and M segment of Batai virus. This is particularly interesting because Ngari virus can be associated with large outbreaks of severe illness in East Africa while its parents are reported to cause rather mild symptoms in humans but more severe symptoms including abortions and teratogenic effects in livestock [37–40].

Among phleboviruses, multiple inter-lineage reassortment events were reported for Rift Valley virus [41] and for severe fever with thrombocytopenia syndrome virus [42, 43]. Moreover, some phleboviruses such as Aguacate [44] or Granada [45] are considered to be heterotypic reassortants. Interestingly, reassortment events have been so far repeatedly reported only for Crimean-Congo hemorrhagic fever virus [46–49] but for no other members of the *Nairoviridae* family.

### Hantaviruses: mostly intra-species (homotypic) reassortment

It is interesting to note that hantaviruses are, in contrast to other bunyaviruses, not transmitted or hosted by arthropods but are tightly associated with small mammals as their reservoir hosts. Based on the concept of Briese et al. [32], this fact should reduce the extent of co-infections and thereby also reduce the probability of reassortment events. Indeed, most of the reports on reassortment in hantaviruses are limited to inter-lineage events within the same virus species usually carried by a single reservoir host (Table 1). The phenomenon of heterotypic reassortment frequently seen in orthobunyaviruses or phleboviruses seems to be very rare or at least could not be well-documented yet.

**Table 1:** Summary of the reported naturally occurring intra-species reassortment events among hantaviruses

Virus	Reassortment scenarios^a^	References
Sin Nombre virus	S_A_M_B_L_ND_	[1]
	Mostly S_A_M_B_L_A_, S_B_M_A_L_B_, rarely S_B_M_A_L_A_, S_A_M_B_L_B_, S_A_M_A_L_B_	[2]
	S_A_M_B_L_ND_	[50]
Puumala virus	S_B_M_A_L_A_, S_A_M_B_L_B_, S_B_M_A_L_B_	[51]
	S_A_M_B_L_A_, S_B_M_A_L_B_	[52]
	All six combinations but mostly S_B_M_A_L_B_ and S_B_M_A_L_A_	[53]
	S_A_M_B_L_ND_	[54]
Dobrava-Belgrade virus	S_A_M_B_L_ND_	[55, 58]
Hantaan virus	S_A_M_A_L_B_	[60]
Seoul virus	S_A_M_B_L_ND_	[61]
Imjin virus	S_A_M_B_L_A_	[62]
Seewis virus	S_A_M_ND_L_B_	[63]

^a^Schematic representation of the reassortment pattern. S, M, and L capital letters stand for the S, M, and L genomic segments. Subscripted A and B letters indicate origin of the given segment to one of the two hypothetical, phylogenetically distinct parents. ND indicates that the origin was not determined

First findings indicating reassortment events in hantaviruses were reported for SNV soon after its discovery. Li et al. [1] analyzed complete S and M segment sequences of two virus isolates from eastern California and found that while their M segment sequences differed from one another by only 1%, the S segments differed by 13%. They concluded reassortment as the most likely explanation for their data. These findings were then confirmed and further extended by the analyses of SNV sequences obtained from deer mice (*Peromyscus maniculatus)*, the principal host of SNV, trapped in Nevada and eastern California. Phylogenetic analyses of all three segments indicated that several segment exchanges were possible but those involving M segment were found most frequently. Conflicting signals in the S and M segment-based phylogenetic trees suggesting reassortment were also found in a more recent study involving SNV sequences obtained from deer mice collected in Colorado, New Mexico, and Montana from 1995 to 2007 [50].

**Fig. 1:**
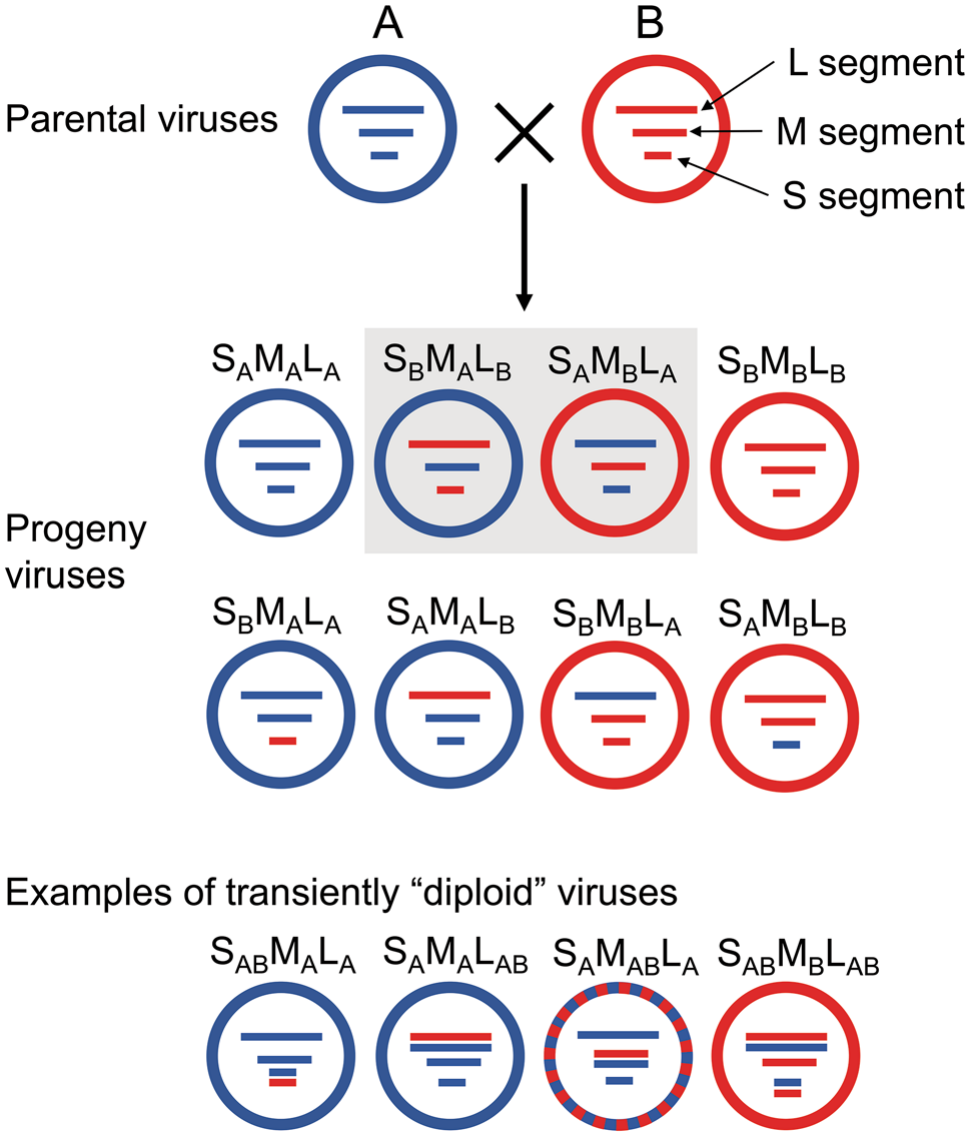
Schematic representation of all potential reassortants resulting from the co-infection of a cell by two hypothetical parental hantaviruses **a** (blue) and **b** (red). Envelope color corresponds to the color of the encoding M segment. S, M, and L capital letters stand for the S, M, and L genomic segments. Subscripted A and B indicate origin of the given segment to one of the two parents. The reassortants generated in the in vitro experiments and also most frequently found among the naturally occurring reassortants are marked by grey background. “Diploid” viruses containing both parental versions of at least one segment were transiently observed in the in vitro experiments. Four examples out of the 13 possible “diploid” patterns are shown in the bottom part of the figure

Occurrence of reassortment events is well documented also for the most common European hantavirus, Puumala virus (PUUV) associated with bank voles (*Myodes glareolus*). In a series of systematic studies performed in central and northern Finland, notably high frequency of reassortment, 19.1–32%, could be observed [51–53]. Moreover, one interesting phenomenon could be noticed. The studies in central Finland identified reassortants between two phylogenetic clusters within the same, Finish lineage [51, 53]. In this case, basically all six possible segment combinations were found and the most common were those schematically designated as S_B_M_A_L_A_, S_A_M_B_L_B_, S_B_M_A_L_B_ where the S, M, and L capital letters stand for the genomic segments and the subscripted A and B letters indicate origin of the given segment to one of the two hypothetical, phylogenetically distinct parents (Fig. 1). In the study from northern Finland, reassortment was detected between more distantly related groups of viruses representing two distinct, previously defined lineages, the Finnish and the North Scandinavian lineage. This fact did not reduce the frequency of reassortment observations, it was actually 32%, the highest value among the three studies. However, of the six possible segment combinations, only two were found, those where both S and L segments originated from the same genetic lineage [52]. This pattern is typically found in the heterotypic reassortment events described for orthobunyaviruses [32] and also with in vitro generated hantavirus reassortants (see below).

In a phylogenetic study focused on PUUV sequences obtained from bank voles captured in Central Europe [54], authors noticed that the strains from eastern Slovakia clustered as expected with the sequences from Bohemian Forrest (Czech Republic) and Bavarian Forrest (Germany) only in the M but not S segment analyses. In the S segment analyses, the Slovak strains surprisingly clustered with the strains found in bank voles from Belgium, France, the south of the Netherlands, and regions in north-western Germany. This topology incongruence indicates the occurrence of a reassortment event. Since only partial sequences were analyzed, homologous recombination cannot be completely ruled out, too. Unfortunately, analysis of only partial sequences is a limitation of most of the studies analyzing sequences obtained directly from the hantavirus reservoir hosts. It seems to be a general consensus that the conflicting signals are interpreted as hints for reassortment but not for homologous recombination.

It is also interesting to note that besides the recent PUUV study [54], indications of reassortment, and/or recombination were reported from eastern Slovakia also for other hantaviruses [55, 56]. This phenomenon might be directly associated with the fact that eastern Slovakia has been involved in several end-glacial colonization routes of rodents. The region is a contact area for three phylogeographic clades of bank voles; the Carpathian, Western, and Eastern clade [57]. The region, therefore, seems to be a melting pot providing ample opportunity for the sympatric occurrence of several virus lineages consequently leading to co-infections followed by reassortment and/or recombination events.

Indications for reassortment events found within *Dobrava-Belgrade orthohantavirus* species, although still regarded as inter-lineage events, perhaps mostly resemble the heterotypic reassortments observed for orthobunyaviruses. In contrast to SNV or PUUV associated with a single reservoir host, DOBV lineages, designated as genotypes [58], are associated with distinct species of the *Apodemus* sp. mice. Saaremaa virus found in striped field mice (*A. agrarius*) on Saaremaa island, Estonia seems to be a reassortant containing M segment clustering within another *A. agrarius*-associated genotype, Kurkino, while its position in the S phylogenetic trees is more ancestral and more closely related with the *A. flavicollis*- and *A. ponticus*-associated Dobrava and Sochi genotypes, respectively. Similar conflicts in tree topologies suggesting genetic reassortment during DOBV evolution have been observed also for the Sochi genotype. In S segment trees, Sochi sequences form a well-supported sister group to Dobrava genotype but form an out-group to all other DOBV strains in M and L segment trees [59].

Analysis of 34 complete genome sequences of HTNV acquired from *A. agrarius* mice captured from 2003 to 2014 in the Republic of Korea indicated occurrence of natural reassortment events in the evolution of HTNV. In addition to the observation of conflicting tree topologies, the authors provided additional evidence of reassortment by application of a whole array of recombination detecting algorithms on artificially concatenated complete sequences of all three segments. Interestingly, in this case, only a pattern schematically designated as S_A_M_A_L_B_ could be found [60]. Differences in the clustering patterns of S and M segment-based phylogenetic tress suggested inter-lineage reassortment also among SEOV lineages in central south China [61].

Indications for reassortment events were recently reported also for the shrew-borne hantaviruses. Imjin virus (MJNV) is a shrew-borne hantavirus identified in the Ussuri white-toothed shrews (*Crocidura lasiura*) in the Republic of Korea and China. The reassortment pattern S_A_M_B_L_A_ was recently identified in a study from the Republic of Korea [62]. Intriguing differences between the L-segment and S-segment phylogenies implying multiple reassortment events were observed also in extensive phylogenetic analysis of *Sorex araneus* shrew-borne Seewis virus [63].

### Heterotypic or “ancient” reassortment events

All the above described reassortment events can be considered as intra- or inter-lineage events between closely related strains of the same virus. In most cases, these events also occur within a single reservoir host. However, there are few reports which describe reassortment events between distinct viruses or phylogenetic incongruences at deep nodes. Two of those reports are originating from south America where numerous rodent species have been identified to harbor unique hantavirus strains. Ape Aime-Itapúa virus (AAIV) was identified in *Akodon montensis* from Paraguay [64]; in the S segment tree, AAIV clusters with Jabora virus (JABV) associated with *A. montensis*. In contrast, in the M segment analyses AAIV shows a strong relationship with Pergamino virus, originally identified in Argentina in *A. azarae* [64, 65]. However, JABV by itself also shows conflicting positions in the S and M segment trees. The whole JABV clade clusters in the M segment tree with Maporal virus associated with the fulvous pygmy rice rat (*Oligoryzomys fulvescens*). However, in the S segment analyses, JABV clusters only with AAIV and occupies the most ancestral position within the South American hantaviruses [65]. These findings indicate that reassortment events involving distantly related hantaviruses are directly connected with or play a role in the processes of host-switching.

Very surprising findings were reported by Zou et al. [66]. Sequence analysis of cell culture isolates originating from *A. agrarius* mice and *Rattus norvegicus* rats revealed that spill over infections of HTNV from its reservoir host, *A. agrarius*, to *R. norvegicus* rats might be quite common. Most unexpectedly, two isolates, originally generated in 1988 from *R. norvegicus* rats, were shown to contain M and L segments of SEOV but the S segment of HTNV. This finding raises the question that, if such reassortants of HTNV and SEOV are possible, what could be their consequences for the public health and why are they not detected more frequently. It remains to be seen whether such viruses remained unnoticed because sequence analyses of more than one segment were not routinely performed until recently or whether those two isolates represent highly exceptional findings.

Recently, Bruges virus as second hantavirus in addition to Nova virus was identified to be harbored by the European mole (*Talpa europaea*). Occurrence of two highly divergent viruses in the same reservoir host shows that at least one of the viruses was involved in host-switching processes. Phylogenetic analyses of all three genomic segments showed tree topology inconsistencies at deep nodes, suggesting that Bruges virus may have emerged from ancient reassortment events. The virus appeared to be most closely related to hantaviruses associated with hosts from the *Muridae* family, to hantaviruses carried by shrews and moles, or showed ancestral position to both these groups in the S, M, and L segment-specific analyses based on complete coding sequences, respectively [67].

### In vitro generated reassortants

Most of the reports claiming occurrence of reassortment in hantaviruses are based on phylogenetic analyses of naturally occurring strains, mainly obtained from the reservoir hosts. The basis for these claims are conflicting tree topologies, sometimes accompanied by more advanced phylogenetic analyses including hypothesis testing or recombination analysis on concatenated sequences. In other words, the claims are based on descriptive bioinformatic analyses showing the reassortment only indirectly. However, there are several reports bringing the ultimate proof that hantaviruses are capable to exchange genome segments during co-infections through in vitro experiments (Table 2).

**Table 2:** Summary of in vitro generated hantavirus reassortants

Parental viruses	Generated reassortants^a^	References
SNV_NMR11_, SNV_CC107_	S_NMR11_M_CC107_L_NMR11_, S_CC107_M_NMR11_L_NMR11_, S_CC107_M_CC1071_L_NMR11_	[68]
SNV, BCCV	S_BCCV_M_SNV_L_BCCV_	[68]
SNV, ANDV	S_SNV_M_ANDV_L_SNV_	[69]
SNV, ANDV	S_SNV_M_ANDV_L_SNV_	[70]
PHV, PUUV	S_PHV_M_PUUV_L_PHV_	[72]
DOBV_SK/Aa_, DOBV_Slo/Af_	S_SK/Aa_M_Slo/Af_L_SK/Aa_, S_Slo/Af_M_SK/Aa_L_Slo/Af_	[73]

*SNV* Sin Nombre virus; *BCCV* Black Creek Canal virus; *ANDV* Andes virus; *PHV* Prospect Hill virus; *PUUV* Puumala virus; *DOBV* Dobrava-Belgrade virus

^a^Schematic representation of the reassortment pattern. S, M, and L capital letters stand for the S, M, and L genomic segments. Subscripted part indicates origin of the given segment to a particular parental virus and is given either as a virus abbreviation or strain name

Rodriguez et al. [68] showed already in 1998 that mixed infection of Vero E6 cells with two distinct strains of SNV can lead to generation of reassortant viruses in 8.5% of 294 progeny plaques tested. Most of the reassortants had the patterns S_A_M_B_L_A_ and S_A_M_B_L_B_. On the other hand, only one virus reassortant was observed among 163 progeny virus plaques from mixed infections between SNV and Black Creek Canal virus (BCCV), an HPS-causing virus from Florida, which has the cotton rat (*Sigmodon hispidus*) as its natural host; the reassortant carried the M segment of SNV and S and L segments of BCCV. Interestingly, in both experiments about 30% of the progeny virus plaques appeared to be transiently diploid, i.e. containing both versions of at least one segment.

Similar experiments were performed with SNV and ANDV by Rizvanov et al. [69]. Again, also diploid viruses were observed (20/337 progeny plaques) and all monoploid reassortant viruses (10/337 progeny plaques) contained the S and L segments of SNV but ANDV M segment. Despite having from ANDV only the M segment, the reassorted virus showed replication efficiency in Vero E6 cells resembling ANDV rather than SNV.

The very same reassortment pattern was achieved also in the study of McElroy et al. [70]. In line with the previously mentioned study [69], the virus containing the S and L segments of SNV and M segment of ANDV (designated SAS) had in vitro growth and plaque morphology characteristics similar to those of ANDV. Most important results were obtained in the in vivo experiment. The SAS reassortant virus was highly infectious and elicited high-titer, ANDV-specific neutralizing antibodies in Syrian hamsters. However, the virus did not cause lethal HPS indicating that the ANDV M genome segment alone is not sufficient to confer the lethal HPS phenotype described for ANDV [71].

Reassortant could be later generated also between a pathogenic PUUV and a non-pathogenic Prospect Hill virus (PHV). The reassortant contained the glycoprotein coding M-segment derived from PUUV and the S and L segments from PHV. The reassortant together with parental viruses were characterized also in terms of their ability to modulate in vitro innate immune responses including induction of type I and type III interferon and interferon-stimulated gene MxA. In all experiments, the reassortant revealed the same characteristic innate antiviral response pattern as PHV, which is considered to be a non-pathogenic hantavirus. These data are not only consistent with the previous studies on SNV and ANDV reassortants but also led the authors to the conclusion that such reassortant viruses carrying M segment of the pathogenic virus together with S and L segments of non-pathogenic virus (such as PHV) could be used as attenuated vaccines [72].

Inspired by the phylogenetic findings of putative reassortment events in DOBV [55], Kirsanovs et al. [73] performed mixed infections and found efficient in vitro reassortment between members of two different DOBV genetic lineages, the weakly virulent DOBV-Aa (nowadays designated as Kurkino genotype) and highly virulent DOBV-Af (Dobrava genotype). High frequency of reassortment was observed. Reassortment patterns were found in 65 out of 207 analyzed progeny clones (31.4%). As in the previous in vitro studies, only reassortant having S and L segments from the same parental virus and exchanged M segment were generated. In this case, both versions (schematically designated as S_A_M_B_L_A_ and S_B_M_A_L_B_ throughout this review) were found. Analogously to the study of Handke et al. [72], the reassortants were (together with the parental viruses) analyzed for the differential induction of innate immune responses in the established cell lines A549 and HuH7. The contrasting phenotypes of the parental viruses were found to be maintained by the reassortants carrying the respective S and L segments of the parental virus and were not influenced by the origin of the M segment.

Also in this reassortment experiment, significantly high proportion of the analyzed clones (65/207; 31.4%) were designated as diploids containing both parental versions of at least one segment [73]. In fact, diploid viruses were observed in all studies on in vitro generated reassortants mentioned above. These findings indicate that the hantavirus assembly process is not tightly controlled and more than three genome segments can be packed into the viral particle. This imperfect segment packaging is perhaps directly involved in the ability of hantaviruses to reassort.

## Conclusions

Although not as frequent as in other arthropod-borne bunyaviruses, reassortment seems to be more common among hantaviruses than initially recognized. The intra- and inter-lineage reassortment between closely related variants seems to occur whenever co-infections of two virus variants are possible due to their sympatric occurrence.

On the other hand, heterotypic reassortment between more distantly related viruses occurs less frequently but seems to play a supporting role in the process of crossing the species barriers and host switching when, e.g., the newly acquired M segment encoding the envelope glycoproteins might help the virus to establish persistent infection in the new host. Topologic incongruences in the deep nodes of the segment specific trees suggest that such events occurred in the past and contributed to the currently recognized hantavirus diversity.

All the reported in vitro reassortment experiments have in common that only reassortants with exchanged M segments could be generated. This finding suggests that a high degree of genetic compatibility including packaging signals and RNA–RNA and/or RNA–protein interactions is required particularly for the S and L segments while the exchange of M segment is better tolerated or is particularly beneficial.

Altogether, the numerous reports on hantavirus reassortment varying from naturally occurring intra-lineage reassortants to in vitro generated inter-species reassortants, as summarized in this review, clearly demonstrate that reassortment events play a significant role in hantavirus evolution. Consequently, it will be highly beneficial to invest in obtaining complete sequences of all three genomic segments in the future studies. It can be assumed that advancement of the next-generation sequencing technologies will generate more high-quality data which are needed to further elaborate the current accumulating evidence of reassortment as a significant driving force in hantavirus evolution.

## References

[1] Li D, Schmaljohn AL, Anderson K, Schmaljohn CS (1995). Complete nucleotide sequences of the M and S segments of two hantavirus isolates from California: evidence for reassortment in nature among viruses related to hantavirus pulmonary syndrome. Virology.

[2] Henderson WW, Monroe MC, St Jeor SC (1995). Naturally occurring *Sin Nombre virus* genetic reassortants. Virology.

[3] Reuter M, Krüger DH (2018). The nucleocapsid protein of hantaviruses: much more than a genome-wrapping protein. Virus Genes.

[4] Cifuentes-Muñoz N, Salazar-Quiroz N, Tischler ND (2014). Hantavirus Gn and Gc envelope glycoproteins: key structural units for virus cell entry and virus assembly. Viruses.

[5] Kukkonen SKJ, Vaheri A, Plyusnin A (2005). L protein, the RNA-dependent RNA polymerase of hantaviruses. Arch Virol.

[6] Gavrilovskaya IN, Shepley M, Shaw R (1998). beta3 Integrins mediate the cellular entry of hantaviruses that cause respiratory failure. Proc Natl Acad Sci USA.

[7] Geimonen E, Neff S, Raymond T (2002). Pathogenic and nonpathogenic hantaviruses differentially regulate endothelial cell responses. Proc Natl Acad Sci USA.

[8] Raftery MJ, Lalwani P, Krautkrämer E (2014). Β2 Integrin mediates hantavirus-induced release of neutrophil extracellular traps. J Exp Med.

[9] Krautkrämer E, Zeier M (2008). Hantavirus causing hemorrhagic fever with renal syndrome enters from the apical surface and requires decay-accelerating factor (DAF/CD55). J Virol.

[10] Choi Y, Kwon Y-C, Kim S-I (2008). A hantavirus causing hemorrhagic fever with renal syndrome requires gC1qR/p32 for efficient cell binding and infection. Virology.

[11] Jin M, Park J, Lee S (2002). Hantaan virus enters cells by clathrin-dependent receptor-mediated endocytosis. Virology.

[12] Ramanathan HN, Jonsson CB (2008). New and Old World hantaviruses differentially utilize host cytoskeletal components during their life cycles. Virology.

[13] Vaheri A, Strandin T, Hepojoki J (2013). Uncovering the mysteries of hantavirus infections. Nat Rev Microbiol.

[14] Mir MA, Duran WA, Hjelle BL (2008). Storage of cellular 5′ mRNA caps in P bodies for viral cap-snatching. Proc Natl Acad Sci.

[15] Garcin D, Lezzi M, Dobbs M (1995). The 5′ ends of Hantaan virus (Bunyaviridae) RNAs suggest a prime-and-realign mechanism for the initiation of RNA synthesis. J Virol.

[16] Ramanathan HN, Chung D-H, Plane SJ (2007). Dynein-dependent transport of the Hantaan virus nucleocapsid protein to the endoplasmic reticulum-Golgi intermediate compartment. J Virol.

[17] Rowe RK, Suszko JW, Pekosz A (2008). Roles for the recycling endosome, Rab8, and Rab11 in hantavirus release from epithelial cells. Virology.

[18] Krüger DH, Schönrich G, Klempa B (2011). Human pathogenic hantaviruses and prevention of infection. Hum Vaccin.

[19] Witkowski PT, Perley CC, Brocato RL (2017). Gastrointestinal tract as entry route for hantavirus infection. Front Microbiol.

[20] Klempa B, Fichet-Calvet E, Lecompte E (2007). Novel hantavirus sequences in Shrew, Guinea. Emerg Infect Dis.

[21] Arai S, Ohdachi SD, Asakawa M (2008). Molecular phylogeny of a newfound hantavirus in the Japanese shrew mole (*Urotrichus talpoides*). Proc Natl Acad Sci USA.

[22] Weiss S, Witkowski PT, Auste B (2012). Hantavirus in bat, Sierra Leone. Emerg Infect Dis.

[23] Shi M, Lin X-D, Chen X (2018). The evolutionary history of vertebrate RNA viruses. Nature.

[24] Hughes a L, Friedman R (2000). Evolutionary diversification of protein-coding genes of hantaviruses. Mol Biol Evol.

[25] Guo W-P, Lin X-D, Wang W (2013). Phylogeny and origins of hantaviruses harbored by bats, insectivores, and rodents. PLoS Pathog.

[26] Bennett SN, Gu SH, Kang HJ (2014). Reconstructing the evolutionary origins and phylogeography of hantaviruses. Trends Microbiol.

[27] Witkowski PT, Drexler JF, Kallies R (2016). Phylogenetic analysis of a newfound bat-borne hantavirus supports a laurasiatheria host association for ancestral mammalian hantaviruses. Infect Genet Evol.

[28] Yanagihara R, Gu SH, Arai S (2014). Hantaviruses: rediscovery and new beginnings. Virus Res.

[29] McDonald SM, Nelson MI, Turner PE, Patton JT (2016). Reassortment in segmented RNA viruses: mechanisms and outcomes. Nat Rev Microbiol.

[30] White MC, Lowen AC (2018). Implications of segment mismatch for influenza A virus evolution. J Gen Virol.

[31] Stenglein MD, Jacobson ER, Chang LW (2015). Widespread recombination, reassortment, and transmission of unbalanced compound viral genotypes in natural arenavirus infections. PLoS Pathog.

[32] Briese T, Calisher CH, Higgs S (2013). Viruses of the family Bunyaviridae: are all available isolates reassortants?. Virology.

[33] Aguilar PV, Barrett AD, Saeed MF (2011). Iquitos virus: a novel reassortant orthobunyavirus associated with human illness in Peru. PLoS Negl Trop Dis.

[34] Saeed MF, Wang H, Suderman M (2001). Jatobal virus is a reassortant containing the small RNA of Oropouche virus. Virus Res.

[35] Goller KV, Höper D, Schirrmeier H (2012). Schmallenberg virus as possible ancestor of Shamonda virus. Emerg Infect Dis.

[36] Nunes MRT, Travassos da Rosa APA, Weaver SC (2005). Molecular epidemiology of group C viruses (Bunyaviridae, Orthobunyavirus) isolated in the Americas. J Virol.

[37] Gerrard SR, Li L, Barrett AD, Nichol ST (2004). Ngari virus is a Bunyamwera virus reassortant that can be associated with large outbreaks of hemorrhagic fever in Africa. J Virol.

[38] Briese T, Bird B, Kapoor V (2006). Batai and Ngari viruses: M segment reassortment and association with severe febrile disease outbreaks in East Africa. J Virol.

[39] Bowen MD, Trappier SG, Sanchez AJ (2001). A reassortant bunyavirus isolated from acute hemorrhagic fever cases in Kenya and Somalia. Virology.

[40] Dutuze MF, Nzayirambaho M, Mores CN, Christofferson RC (2018). A review of Bunyamwera, Batai, and Ngari viruses: understudied orthobunyaviruses with potential One Health implications. Front Vet Sci.

[41] Bird BH, Khristova ML, Rollin PE (2007). Complete genome analysis of 33 ecologically and biologically diverse Rift Valley fever virus strains reveals widespread virus movement and low genetic diversity due to recent common ancestry. J Virol.

[42] Lv Q, Zhang H, Tian L (2017). Novel sub-lineages, recombinants and reassortants of severe fever with thrombocytopenia syndrome virus. Ticks Tick Borne Dis.

[43] Liu J-W, Zhao L, Luo L-M (2016). Molecular evolution and spatial transmission of severe fever with thrombocytopenia syndrome virus based on complete genome sequences. PLoS ONE.

[44] Palacios G, da Rosa AT, Savji N (2011). Aguacate virus, a new antigenic complex of the genus Phlebovirus (family Bunyaviridae). J Gen Virol.

[45] Collao X, Palacios G, de Ory F (2010). Granada virus: a natural phlebovirus reassortant of the sandfly fever Naples serocomplex with low seroprevalence in humans. Am J Trop Med Hyg.

[46] Hewson R, Gmyl A, Gmyl L (2004). Evidence of segment reassortment in Crimean-Congo haemorrhagic fever virus. J Gen Virol.

[47] Zhou Z, Deng F, Han N (2013). Reassortment and migration analysis of Crimean-Congo haemorrhagic fever virus. J Gen Virol.

[48] Goedhals D, Bester PA, Paweska JT (2014). Next-generation sequencing of southern African Crimean-Congo haemorrhagic fever virus isolates reveals a high frequency of M segment reassortment. Epidemiol Infect.

[49] Lukashev AN, Klimentov AS, Smirnova SE (2016). Phylogeography of crimean congo hemorrhagic fever virus. PLoS ONE.

[50] Black WC, Doty JB, Hughes MT (2009). Temporal and geographic evidence for evolution of Sin Nombre virus using molecular analyses of viral RNA from Colorado, New Mexico and Montana. Virol J.

[51] Razzauti M, Plyusnina A, Henttonen H, Plyusnin A (2008). Accumulation of point mutations and reassortment of genomic RNA segments are involved in the microevolution of Puumala hantavirus in a bank vole (*Myodes glareolus*) population. J Gen Virol.

[52] Razzauti M, Plyusnina A, Sironen T (2009). Analysis of Puumala hantavirus in a bank vole population in northern Finland: evidence for co-circulation of two genetic lineages and frequent reassortment between strains. J Gen Virol.

[53] Razzauti M, Plyusnina A, Henttonen H, Plyusnin A (2013). Microevolution of Puumala hantavirus during a complete population cycle of its host, the bank vole (Myodes glareolus). PLoS ONE.

[54] Szabó R, Radosa L, Ličková M (2017). Phylogenetic analysis of Puumala virus strains from Central Europe highlights the need for a full-genome perspective on hantavirus evolution. Virus Genes.

[55] Klempa B, Schmidt HA, Ulrich R (2003). Genetic interaction between distinct Dobrava hantavirus subtypes in *Apodemus agrarius* and *A. flavicollis* in nature. J Virol.

[56] Sibold C, Meisel H, Krüger DH (1999). Recombination in Tula hantavirus evolution: analysis of genetic lineages from Slovakia. J Virol.

[57] Filipi K, Marková S, Searle JB, Kotlík P (2015). Mitogenomic phylogenetics of the bank vole *Clethrionomys glareolus*, a model system for studying end-glacial colonization of Europe. Mol Phylogenet Evol.

[58] Klempa B, Avsic-Zupanc T, Clement J (2013). Complex evolution and epidemiology of Dobrava-Belgrade hantavirus: definition of genotypes and their characteristics. Arch Virol.

[59] Klempa B, Tkachenko EA, Dzagurova TK (2008). Hemorrhagic fever with renal syndrome caused by 2 lineages of Dobrava hantavirus, Russia. Emerg Infect Dis.

[60] Kim J-A, Kim W-K, No JS (2016). Genetic diversity and reassortment of Hantaan virus tripartite RNA genomes in nature, the Republic of Korea. PLoS Negl Trop Dis.

[61] Liu J, Liu D-Y, Chen W (2012). Genetic analysis of hantaviruses and their rodent hosts in central-south China. Virus Res.

[62] Lee S-H, Kim W-K, No JS (2017). Dynamic circulation and genetic exchange of a shrew-borne hantavirus, Imjin virus, in the Republic of Korea. Sci Rep.

[63] Ling J, Smura T, Tamarit D (2018). Evolution and postglacial colonization of Seewis hantavirus with Sorex araneus in Finland. Infect Genet Evol.

[64] Chu YK, Milligan B, Owen RD (2006). Phylogenetic and geographical relationships of hantavirus strains in eastern and western Paraguay. Am J Trop Med Hyg.

[65] Chu YK, Owen RD, Jonsson CB (2011). Phylogenetic exploration of hantaviruses in paraguay reveals reassortment and host switching in South America. Virol J.

[66] Zou Y, Hu J, Wang Z-X (2008). Genetic characterization of hantaviruses isolated from Guizhou, China: evidence for spillover and reassortment in nature. J Med Virol.

[67] Laenen L, Vergote V, Kafetzopoulou LE (2018). A novel hantavirus of the European mole, Bruges virus, is involved in frequent Nova virus coinfections. Genome Biol Evol.

[68] Rodriguez LL, Owens JH, Peters CJ, Nichol ST (1998). Genetic reassortment among viruses causing hantavirus pulmonary syndrome. Virology.

[69] Rizvanov A, Khaiboullina SF, St Jeor S (2004). Development of reassortant viruses between pathogenic hantavirus strains. Virology.

[70] McElroy a K, Smith JM, Hooper JW, Schmaljohn CS (2004). Andes virus M genome segment is not sufficient to confer the virulence associated with Andes virus in Syrian hamsters. Virology.

[71] Hooper JW, Larsen T, Custer DM, Schmaljohn CS (2001). A lethal disease model for hantavirus pulmonary syndrome. Virology.

[72] Handke W, Oelschlegel R, Franke R (2010). Generation and characterization of genetic reassortants between Puumala and Prospect Hill hantavirus in vitro. J Gen Virol.

[73] Kirsanovs S, Klempa B, Franke R (2010). Genetic reassortment between high-virulent and low-virulent Dobrava-Belgrade virus strains. Virus Genes.

